# Effects of Decreasing Dietary Crude Protein Level on Growth Performance, Nutrient Digestion, Serum Metabolites, and Nitrogen Utilization in Growing Goat Kids (*Capra.* *hircus*)

**DOI:** 10.3390/ani10010151

**Published:** 2020-01-16

**Authors:** Wen Zhu, Wei Xu, Congcong Wei, Zijun Zhang, Chunchao Jiang, Xingyong Chen

**Affiliations:** 1 College of Animal Science and Technology, Anhui Agricultural University, Hefei 230036, China; zhuwen@ahau.edu.cn (W.Z.); xuwei6494@163.com (W.X.); wcc17681322537@163.com (C.W.); zhangzijun@ahau.edu.cn (Z.Z.); 2 Luan Lvjie Animal Husbandry Co., Ltd., Luan 237000, China; lvjiemuye@126.com

**Keywords:** crude protein, growth performance, nutrient digestion, nitrogen utilization, Anhui white goat

## Abstract

**Simple Summary:**

Reducing the dietary protein content could potentially reduce losses of nitrogen from ruminant farms and mitigate pressure on the protein ingredient supply. However, there is little information in the literature on the effect of low-protein diets in growing Anhui white goat kids. We demonstrated that decreasing the dietary crude protein level in Anhui white goat kids affected growth performance, improved nitrogen utilization, and reduced environmental nitrogen pollution. The key finding of this study was that a diet containing 13.4% crude protein supplied adequate protein to improve nitrogen utilization in white goat kids without any adverse effect on growth performance.

**Abstract:**

The effects of decreasing dietary crude protein (CP) level on growth performance, nutrient digestion, serum metabolites, and nitrogen utilization in growing goat kids were investigated in the current study. Thirty-six male Anhui white goat kids were randomly assigned to one of three CP content diets: 14.8% (control), 13.4%, and 12.0% of dry matter, respectively. Diets were isoenergetic. The experiment lasted for 14 weeks, with the first two weeks being for adaptation. Results showed that the low-CP diet decreased average daily gain, feed efficiency, digestibility of dry matter, organic matter, crude protein, and fiber. No significant changes were observed in dry-matter intake. With a decrease in dietary CP level, fecal nitrogen excretion (% of nitrogen intake) increased linearly, whereas CP intake, blood urea nitrogen, urinary nitrogen excretion (% of nitrogen intake), and total nitrogen excretion (% of nitrogen intake) decreased. Serum glucose concentration decreased, while concentrations of low-density lipoproteins and non-esterified fatty acids increased with the low-CP diet. In conclusion, decreasing the dietary CP level decreased goats’ nitrogen excretion, but with restrictive effects on growth performance. A diet containing 13.4% CP is optimal for reducing nitrogen excretion without any adverse effect on growth performance of Anhui white goat kids. This concentration is 1.4% points lower than the NRC recommendations and thus is also environmentally beneficial on the input side because it decreases the use of feed (soy) protein.

## 1. Introduction

In ruminants, the part of microbial degradable dietary nitrogen (N) is converted to ammonia in the rumen [1]. The excessive ammonia absorbed through the rumen wall into the blood converts to urea in the liver, which is excreted in the urine [2]. Nitrogen excretion results in water pollution, soil acidification, and formation of fine particles [3]. As an important source of food, fiber, and economic security [4], goats are an important ruminant species, especially in developing countries. As the global population of goats is approximately 654 million the goat industry should take responsibility for N excretions [5]. According to previous studies, reducing dietary protein level is an effective way to reduce N excretion [6,7].

China has 140 million goats, which account for 21.4% of the global population [5]. Anhui white goat is a local meat-type breed in China which is mainly distributed along Yellow River and Huai River and well-known for its excellent production performance, especially for high fertility [8]. In recent years, chevon (young goat meat) consumption is increasing due to its low content of fat and cholesterol, and favorable nutritive characteristics [9], and Anhui white goats play an increasingly important role in the meat market, especially in the central and southern regions of China. At the same time, high-quality protein sources, such as soybean meal for livestock remain in short supply in China. For example, in 2016, 8.391 million tons of soybean were imported from foreign countries, accounting for more than 26% of the world’s soybean production [10]. Moreover, protein is the most expensive component of goat rations, and over-feeding of protein will be wasted as ammonia [11]. In order to reduce avoidable losses of N from ruminant farms, mitigate pressure on the protein ingredient supply, and improve profitability of goat fattening, it is necessary to feed low-crude-protein (CP) diets to Anhui white goats.

Although the recommendations for CP (%) in diets for fattening goats in different feeding standards are based on the requirements of rumen microorganisms and the host animal [12,13], a comparison of these standards revealed that the CP recommendation for a 15 kg body weight goat with 150 g/d average daily gain (ADG) variety greatly. These differences may be attributed to differences in breed, feed digestibility, and feeding conditions. In China, the range of CP concentrations used in goat fattening varies a lot, and the values used in large-scale farms are close to the NRC recommendations [14,15].

Therefore, the objectives of this study were to investigate the effect of decreasing CP input on growth performance, nutrient digestion, serum metabolites, and nitrogen utilization in the Chinese goat (*Capra. hircus*) sector.

## 2. Materials and Methods

### 2.1. Animals, Diets, and Experimental Design

All experimental protocols were approved by the Animal Care Committee, Anhui Agriculture University, Hefei, China. Thirty-six male Anhui white goat kids (16.0 ± 1.35 kg initial body weight (BW)) were balanced by BW and then randomly allocated to one of 3 experimental dietary treatments (n = 12).

Diets were formulated to be isoenergetic and contained 3 levels of CP (14.8%, 13.4%, and 12.0% of dry matter (DM)). The content of metabolizable energy was 10.5 MJ/kg DM for all diets, with a concentrate to forage ratio of 50:50 (DM basis). The ingredients and composition of the experimental diets are presented in Table 1. The control diet (14.8% CP) used in this experiment was formulated based on the requirements of local indigenous goats (BW 15 kg, ADG 100–150 g/d) according to the NRC recommendations for goats [13]. The other two diets were formulated to meet 90% (13.4% CP) and 80% (12.0% CP) of the CP requirement, respectively.

Animals were fed a total mixed ration in pelleted form, twice daily, at 06:30 and 18:30 h, with approximately 10% feed refusal. The pelleting machine (model: SL-280B) was purchased from Jinta Tongyong Equipment Co., Ltd. (Zhejiang, China). During the pelleting process, steam was added, and the exit temperature was set at 75 °C. The machine yielded an average of 1000 kg DM/h, with a pellet diameter of 2.5 mm.

Animals were individually housed in pens (1.4 m × 1.4 m) with wooden slatted floors. The feeding trial lasted 14 weeks, consisting of 2 weeks for adaption, followed by 12 experimental weeks. The experimental diets were already fed during adaptation. Animals had free access to drinking water throughout the whole experimental period.

### 2.2. Sampling and Measurement

Feeds offered and refused by individual kids was recorded daily before the morning feeding throughout the feeding trial. Body weight was measured individually on 2 consecutive days, with 28-day intervals in week 0, 4, 8, and 12, before feeding. Data were used to calculate average daily gain (ADG). Kids′ feed efficiency (FE) was calculated as the ratio of weight gain to dry-matter intake (DMI).

Rectal fecal samples were collected on days 1, 2, and 3 of week 12 from each goat kid before feeding. Samples of daily feed offer, orts, and feces were stored frozen until the end of the 12-week period and then pooled by kids for the whole experimental period and dried in a forced-air oven at 65 °C for 48 h. Subsamples were subsequently stored in sealed plastic containers at −20 °C for chemical analysis. Before analysis and dried samples were ground through a 1 mm screen in a Cyclotec mill (Tecator 1093; Tecator AB, Hoganas, Sweden). The concentration of DM (method 924.05), organic matter (OM, method 942.05), and CP (method 988.05) was determined according to [16]. Concentrations of neutral detergent fiber (NDF) and acid detergent fiber (ADF) were determined according to [17]. Concentrations of acid-insoluble ash in samples of the feed offer, refusals, and feces were analyzed according to [18].

Blood samples (5 mL) were collected from the jugular vein of each kid into test tubes at approximately 3 h after morning feeding on the 7th day of week 4, 8, and 12. Serum was obtained after centrifugation (3000× *g*, 15 min) and then stored at −20 °C for later analysis. Concentrations of total protein, albumin, blood urea nitrogen (BUN), glucose, total cholesterol, aspartate aminotransferase (AST), alanine aminotransferase (ALT), alkaline phosphatase (ALP), total bilirubin, creatinine, triglyceride (TG), high-density lipoprotein (HDL), low-density lipoprotein (LDL), and non-esterified fatty acids (NEFA) were determined with commercial kits (Beijan· Xinchuangyuan, Beijing, China), using the Toshiba 120 auto biochemistry instrument (Toshiba, Tokyo, Japan). Globulin was calculated by the difference between total protein and albumin.

Spot urine samples were collected twice daily before each feeding, using a commercial urine receptor (Hengkang, Hebei, China) on days 1, 2, and 3 of week 12. The daily fresh urine samples were pooled by kid; 20 mL subsamples were acidified immediately, with an equal volume of 6 mol/L HCl, to prevent ammonia volatilization and stored at −20 °C until analysis. At the end of the experiment, all urine samples were thawed at 25 °C and filtered through Whatman no. 1 filter paper. The filtrates were then analyzed for urea N, according to the colorimetric method [19], and creatine was analyzed by using a picric acid assay [20]. Creatinine has been used as a marker to estimate urine volume and was assumed to be excreted at a rate of 0.334 mmol/kg BW^0.75^ per day [21].

### 2.3. Statistical Analysis

Statistical analyses were carried out by using SAS software (version 9.3, SAS Institute Inc., Cary, NC, USA). Data for DMI and serum metabolites were analyzed by using PROC MIXED with the covariance type AR (1) for repeated measures. The statistical model included week, treatment, and treatment × week interaction as fixed effects, and goat as random effect. Means were separated by using the PDIFF option in the LSMEANS statement.

Data on ADG, FE, nutrient digestion, and nitrogen balance parameters were analyzed by using PROC GLM. The statistical model was the same as indicated above, except that week and treatment × week interactions were omitted.

Linear and quadratic effects of treatment were tested for all data by orthogonal polynomial contrasts. Results are reported as LSMEANS. Significant difference was declared at *p* ≤ 0.05.

## 3. Results

### 3.1. Intake, Growth Performance, and Nutrient Digestibility

The CP level of the diet had no significant effect on DMI (*p* > 0.05) (Table 2). The ADG and FE decreased linearly (*p* < 0.05) with decreasing CP concentration of the diet. Goats fed the 12.0% CP diet had a significantly lower ADG and FE than goats fed the 14.8% and 13.4% CP diet (*p* < 0.05). When calculated based on metabolic weight (BW^0.75^), the OM intake of goats fed the 12.0% CP diet was higher than of goats fed the 14.8% CP diet (*p* < 0.05), with no difference between 13.4% CP and 12.0% CP or 14.8% CP (*p* > 0.05); goats fed the 14.8% CP diet had a higher CP intake than goats fed the 13.4% CP and 12.0% CP diet (*p* < 0.05). Intake of NDF and ADF was not affected by CP level (*p* > 0.05). Total tract apparent digestibility of DM and CP decreased linearly with decreasing CP concentration of diet (*p* < 0.05); goats fed the 12.0% CP diet had a significant lower total tract apparent digestibility of OM and ADF than goats fed the 13.4% CP diet (*p* < 0.05), with no difference between 14.8% CP and 12.0% CP or 13.4% CP (*p* > 0.05). The total tract apparent digestibility of ADF in goats fed the 12.0% CP diet was lower than that of 13.4% CP diet (*p* < 0.05), with the 14.8% CP diet occupying an intermediate rank.

### 3.2. Serum Metabolites

Concentrations of total protein, albumin, globulin, cholesterol, AST, ALP, triglyceride, and HDL were not affected by diet treatment (*p* > 0.05) (Table 3). Concentrations of BUN and glucose decreased linearly with decreasing CP concentration of the diet (*p* < 0.05). Concentrations of total bilirubin, ALT, and creatinine increased linearly with the decreasing CP concentration of the diet (*p* < 0.05). Concentrations of LDL in goats fed the 14.8% CP diet were lower than in goats fed the 13.4% CP and 12.0% CP diet (*p* < 0.05). Concentrations of NEFA increased linearly with decreasing dietary CP concentration (*p* < 0.05).

### 3.3. Nitrogen Balance

The N intake, fecal N excretion, urinary N excretion, total N excretion, and N retention, expressed in g/d, decreased linearly with decreasing CP level of the diet (*p* < 0.05) (Table 4). When expressed as a proportion of N intake, the fecal N excretion increased, while the urinary N excretion decreased for goats receiving the 12.0% CP diet (*p* < 0.05). Total N excretion expressed as a proportion of N intake decreased, while N retention increased with decreasing CP concentration of the diet (*p* < 0.05).

## 4. Discussion

In the present study, DMI was not affected by dietary CP concentration, and similar results were reported by [22]. The ADG and FE were significantly influenced by the CP level, which is consistent with the results of [23], who also reported a decreased ADG and FE in Hainan goats fed diets with decreasing CP levels. This may be due to the fact that goats aged three to six months require high amounts of protein to develop muscle mass, and the lower ADG and FE obtained with decreasing CP level likely resulted from the lower nutrient digestion. In the present study, the 12.0% CP diet seemed to limit the growth performance of Anhui white goats. That decreasing protein content of the diet’s compromising daily weight gain was also observed in finishing Charolais bulls [24]. It is of great importance to formulate diets that foster the digestibility and utilization of feed stuff [25]. To our knowledge, few studies have investigated the effect of a reduction of the dietary protein level on total tract apparent digestibility of nutrients in growing goat kids. In a trial using dairy cows, different dietary protein levels affected the apparent total tract digestibility of DM, OM, CP, ADF, and NDF [26], which is consistent with the results of the present study. However, decreasing CP levels (14.2%, 12.3%, and 10.2%) did not significantly affect DM, OM, CP, NDF, and ADF digestibility in Holstein bulls [27]. The disparity in effects of dietary crude protein level on total tract apparent digestibility of nutrients may be due to differences in experimental conditions. The digestibility of nutrients reflects the activity of microorganisms in rumen [25]; goats fed the 12.0% CP diet showed lower nutrient digestibility than the other two groups, resulting in reduced growth performance. A reason could be that the lower dietary CP level impaired optimal ruminal microbial growth.

Serum parameters are important indices reflecting the health of an animal and its nutritional status [28]. In ruminants, dietary protein is degraded to ruminal ammonia-nitrogen; the BUN [2] and urinary N [29] arise largely from excess ammonia. In the present study, BUN concentration decreased linearly with a decrease in dietary CP level. Decreased BUN concentrations were observed in goats fed low-protein diets [30], as well as in other ruminants [31,32]. The reduced BUN in goats on a low dietary CP level indicates a negative effect of diet on rumen ammonia-nitrogen concentration. Blood glucose concentration is mainly regulated by hepatic gluconeogenesis in ruminants [33]. Decreased blood glucose concentration was observed in growing goats kept on a reduced N diet [34]. These findings are consistent with our results and point to a decrease of hepatic gluconeogenesis. The serum concentrations of triglycerides, HDLC, LDL, and NEFA reflect the state of lipid metabolism and transfer [35]. Glucose is involved in the energy metabolism and synthesis pathways of all mammalian cell types [36]. Increasing serum concentrations of LDL and NEFA may be the result of decreasing glucose concentrations in goats fed low-CP diets. Higher ALT levels in goats fed low protein diets did not indicate liver damage since the concentrations of serum enzymes were within the normal ranges of 7–30 IU/L [37]. A significant increase in serum creatine concentrations in goats fed a low-CP diet in comparison with goats fed a high-CP diet were reported [38], which is similar to the findings of the present experiment. Further studies are needed to determine why a low-CP diet increases serum creatine concentration. Serum AST concentration can be used as an indicator of tissue necrosis in ruminants [39]. The serum concentrations of total protein, albumin, globulin, cholesterol, AST, ALP, triglyceride, and HDL were not affected by the low-CP diet, which suggests that no health problems occurred in the present study.

Effects of decreasing dietary CP levels on N balance in ruminants were determined in many studies. For example, urinary urea excretion (% of N intake) was strongly reduced, whereas fecal N excretion (% of N intake) was not affected in lactating cows and goats with restricted N intake (cows: 19% vs. 26% CP; goats: 11% vs. 17% CP) [40]. In another study, it was reported that urinary urea excretion (% of N intake) was significantly reduced, whereas fecal N excretion (% of N intake) was not influenced when Thai-indigenous beef cattle received 10% vs. 12% CP diets [41]. Nitrogen excretion by livestock via feces and urine leads to the pollution of air and ground water, whereby urea accounts for the major proportion of renal N excretion in ruminants [40]. In the present study, urinary urea excretion (% of N intake) decreased, whereas fecal N excretion (% of N intake) increased in the low-CP diet, whereby the increase of fecal N was likely due to the decreased CP digestibility. A significantly reduced urinary urea excretion (% of N intake) was also observed in N-restricted goats (9% vs. 17% CP) [38]. The lower urinary urea excretion in a low-protein diet is most likely due to increased urea reabsorption in the kidney via an upregulation of urea transporter-A1 mRNA expression [38]. In the present study, high N retention (% of N intake) in goats fed the low-CP diet led to low-urinary-urea excretion. Urinary N is rapidly converted to ammonia and volatilized into the atmosphere, whereas fecal N is converted to ammonia at a much slower rate [42]. Therefore, the decreased urinary N excretion observed with the low-CP diet could reduce environmental N losses. The amount of excreted N reflects protein utilization and N deposition efficiency; therefore, the lower total N excretion (% of N intake) of goats receiving a low-CP diet indicates that such a diet has the potential to reduce environmental N pollution.

## 5. Conclusions

The current study has shown that decreasing dietary CP level decreases goats’ N excretion, thereby reducing environmental N pollution but restricting growth performance. A diet containing 13.4% CP supplies an adequate amount of protein for improved N utilization in Anhui white goat kids, without any adverse effect on growth performance. This concentration is 1.4% points lower than the NRC recommendations and is also environmentally beneficial on the input side, because it decreases the use of feed (soy) protein.

## Figures and Tables

**Table 1:** Ingredients and chemical composition of the experimental diets.

Item	Dietary Crude Protein level ^1^, % DM		
	14.8	13.4	12.0
	**Ingredient, % of DM**		
Ground corn grain	13.0	17.0	21.0
Soybean meal, 43.5% CP ^2^	24.0	20.0	16.0
Wheat bran	7.5	7.5	7.5
Sodium bicarbonate	1.0	1.0	1.0
Salt	1.0	1.0	1.0
Dicalcium phosphate	0.5	0.5	0.5
Calcium carbonate	1.0	1.0	1.0
Premix ^3^	1.0	1.0	1.0
Peanut vine ^4^	28.0	28.0	28.0
Chinese wild rye ^5^	22.0	22.0	22.0
	**Chemical Composition, % of Dry Matter**		
Organic matter	86.3	87.6	88.3
Crude protein	14.8	13.4	12.0
Neutral detergent fiber	46.4	45.6	44.8
Acid detergent fiber	31.8	31.5	31.2
Ether extract	3.74	3.62	3.27
Ash	13.7	12.4	11.7
Metabolizable energy ^6^, MJ/Kg DM	10.5	10.5	10.5

^1^ Treatments 1 to 3 consisted of total mixed ration pellets containing 14.8%, 13.4%, and 12.0% crude protein, respectively. ^2^ Soybean meal contained 89.5% dry matter, 43.5% crude protein, 28.2% neutral detergent fiber, and 10.5% acid detergent fiber. ^3^ Formulated to provide (per kg of dry matter) 600,000 IU of vitamin A, 80,000 IU of vitamin D_3_, 5000 IU of vitamin E, 8000 mg of Zn, 60 mg of Se, 200 mg of I, 9400 mg of Fe, 72 mg of Co, 10,400 mg of Mn, and 1600 mg of Cu. ^4^ Peanut vine contained 91.3% dry matter, 7.17% crude protein, 57.2% neutral detergent fiber, and 50.5% acid detergent fiber on dry matter basis. ^5^ Chinese wild rye contained 89.6% dry matter, 6.7% crude protein, 67.5% neutral detergent fiber, and 38.2% acid detergent fiber on dry-matter basis. ^6^ Calculated according to nutrient requirements of small ruminants [13].

**Table 2:** Effects of low-protein diets on dry matter intake (DMI), average daily gain (ADG), feed efficiency (FE), and apparent total-tract digestibility of nutrients in growing goat kids.

	Crude Protein Level ^1^			SEM	*p*-Value ^2^		
	14.8%	13.4%	12.0%		T	L	Q
DMI, g/d	657.5	664.9	672.9	15.21	0.78	0.48	0.99
ADG, g/d	114 ^a^	116 ^a^	97.3 ^b^	5.24	0.04	0.04	0.12
FE	0.173 ^a^	0.174 ^a^	0.145 ^b^	0.0079	0.02	0.02	0.12
**Intake, per kg Metabolic Weight (BW^0.75^)**							
OM, g/d	49.6 ^b^	50.8 ^a,b^	54.4 ^a^	1.26	<0.001	0.02	0.352
CP, g/d	8.51 ^a^	7.77 ^b^	7.40 ^b^	0.182	<0.001	<0.001	0.301
NDF, g/d	26.7	26.4	27.7	0.61	0.57	0.64	0.87
ADF, g/d	18.3	18.2	19.2	0.43	0.12	0.25	0.65
**Total Tract Apparent Digestibility, %**							
DM	61.2 ^a^	60.5 ^a,b^	56.9 ^b^	0.97	0.004	0.002	0.22
OM	64.5 ^a,b^	65.9 ^a^	62.6 ^b^	0.85	0.02	0.11	0.03
CP	67.5 ^a^	66.3 ^a^	64.1 ^b^	0.85	<0.001	<0.001	0.81
NDF	55.3 ^a^	57.0 ^a^	52.6 ^b^	1.08	0.02	0.08	0.02
ADF	43.4 ^a,b^	46.3 ^a^	41.6 ^b^	1.32	0.04	0.34	0.02

Means within a row with different letters differ significantly (*p* < 0.05). ^1^ BW: body weight; DM: dry matter; OM: Organic matter; CP: Crude protein; NDF: Neutral detergent fiber; ADF: Acid detergent fiber. ^2^ T: Treatment; L: Linear; Q: Quadratic.

**Table 3:** Effects of low-protein diet on serum metabolites in growing goat kids.

Variable	Crude Protein Level			SEM	*p*-Value ^1^		
	14.8%	13.4%	12.0%		T	L	Q
Total protein, g/L	78.6	77.6	79.4	1.17	0.534	0.606	0.322
Albumin, g/L	34.8	35.7	35.4	0.41	0.262	0.253	0.240
Globulin g/L	43.9	41.9	43.9	1.21	0.399	0.997	0.177
Blood urea nitrogen, mmol/L	7.31 ^a^	6.80 ^b^	6.29 ^c^	0.253	0.024	0.160	0.017
Glucose, mmol/L	3.02 ^a^	2.86 ^a,b^	2.60 ^b^	0.067	<0.01	<0.01	0.504
Total cholesterol, mmol/L	2.23	2.54	2.51	0.112	0.130	0.093	0.25
Aspartate aminotransferase, U/L	85.1	87.9	90.1	3.60	0.62	0.33	0.96
Alanine transaminase, U/L	18.7 ^b^	19.4 ^a,b^	20.7 ^a^	0.67	0.04	0.04	0.70
Alkaline phosphatase, U/L	360	358	386	13.5	0.252	0.912	0.09
Total bilirubin, μmol/L	0.712 ^b^	0.780 ^a,b^	1.042 ^a^	0.0297	<0.01	<0.01	<0.01
Creatinine, μmol/L	43.8 ^b^	52.1 ^a^	52.4 ^a^	1.54	<0.01	<0.01	0.04
Triglyceride, mmol/L	0.345	0.329	0.322	0.0162	0.593	0.331	0.781
High-density lipoprotein, mmol/L	1.09	1.10	1.19	0.035	0.09	0.04	0.433
Low-density lipoprotein, mmol/L	0.846 ^b^	1.00 ^a^	0.960 ^a^	0.0373	0.0121	0.0339	0.0309
Non-esterified fatty acid, mmol/L	0.294 ^c^	0.399 ^b^	0.486 ^a^	0.0159	<0.01	<0.01	0.639

Means within a row with different letters differ significantly (*p* < 0.05). ^1^ T: Treatment; L: Linear; Q: Quadratic.

**Table 4:** Effects of low-protein diet on nitrogen balance in growing goat kids.

Variable	Crude Protein Level			SEM	*p*-Value ^1^		
	14.8%	13.4%	12.0%		T	L	Q
Nitrogen intake, g/d	15.6 ^a^	14.3 ^b^	12.9 ^c^	0.33	<0.001	<0.001	0.980
**Fecal Nitrogen Excretion**							
g/d	5.07 ^a^	4.82 ^a,b^	4.63 ^b^	0.101	0.0105	0.0278	0.753
% of nitrogen intake	32.5 ^b^	33.7 ^a,b^	35.9 ^a^	0.85	<0.001	<0.001	0.893
**Urinary Nitrogen Excretion**							
g/d	6.74 ^a^	5.81 ^b^	4.87 ^c^	0.169	<0.001	<0.001	0.458
% of nitrogen intake	43.2 ^a^	40.6 ^b^	37.8 ^c^	0.92	<0.001	<0.001	0.279
**Total Nitrogen Excretion**							
g/d	11.8 ^a^	10.6 ^b^	9.51 ^c^	0.258	<0.001	<0.001	<0.001
% of nitrogen intake	75.7 ^a^	74.3 ^a,b^	73.7 ^b^	1.81	<0.001	<0.001	0.586
**Nitrogen Retention**							
g/d	3.79 ^a^	3.68 ^a^	3.39 ^b^	0.104	0.0214	0.0451	0.879
% of nitrogen intake	24.3 ^b^	25.7 ^a^	26.3 ^a^	0.42	<0.001	<0.001	0.648

Means within a row with different letters differ significantly (*p* < 0.05). ^1^ T: Treatment; L: Linear; Q: Quadratic.
